# Structural Determinants of the Neuronal Glycine Transporter 2 for the Selective Inhibitors ALX1393 and ORG25543

**DOI:** 10.1021/acschemneuro.0c00602

**Published:** 2021-05-18

**Authors:** Cristina Benito-Muñoz, Almudena Perona, Raquel Felipe, Gonzalo Pérez-Siles, Enrique Núñez, Carmen Aragón, Beatriz López-Corcuera

**Affiliations:** † Departamento de Biología Molecular, Universidad Autónoma de Madrid, 28049 Madrid, Spain; ‡ Centro de Biología Molecular “Severo Ochoa” Consejo Superior de Investigaciones Científicas, Universidad Autónoma de Madrid, 28049 Madrid, Spain; § Departamento de Química en Ciencias Farmacéuticas, Universidad Complutense de Madrid, 28040 Madrid, Spain; ∥ IdiPAZ-Hospital Universitario La Paz, Universidad Autónoma de Madrid, 28049 Madrid, Spain

**Keywords:** neuronal glycine transporter 2, glycinergic neurotransmission, pain, inhibitor binding, ALX1393, ORG25543

## Abstract

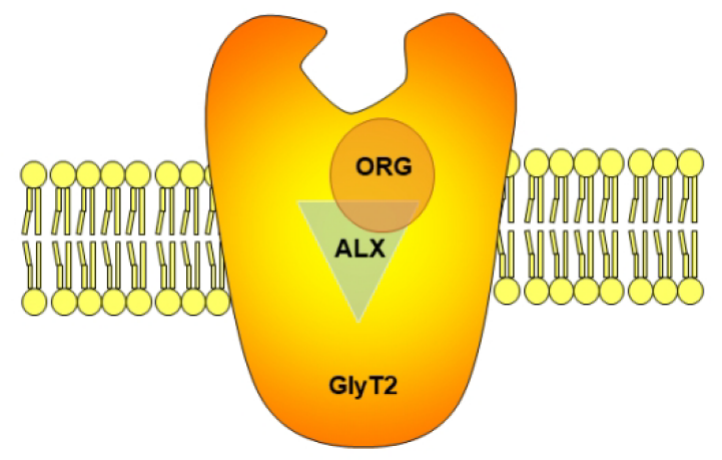

The neuronal glycine transporter GlyT2 modulates inhibitory glycinergic neurotransmission by controlling the extracellular concentration of synaptic glycine and the supply of neurotransmitter to the presynaptic terminal. Spinal cord glycinergic neurons present in the dorsal horn diminish their activity in pathological pain conditions and behave as gate keepers of the touch-pain circuitry. The pharmacological blockade of GlyT2 reduces the progression of the painful signal to rostral areas of the central nervous system by increasing glycine extracellular levels, so it has analgesic action. *O*-[(2-benzyloxyphenyl-3-fluorophenyl)methyl]-l-serine (ALX1393) and *N*-[[1-(dimethylamino)cyclopentyl]methyl]-3,5-dimethoxy-4-(phenylmethoxy)benzamide (ORG25543) are two selective GlyT2 inhibitors with nanomolar affinity for the transporter and analgesic effects in pain animal models, although with deficiencies which preclude further clinical development. In this report, we performed a comparative ligand docking of ALX1393 and ORG25543 on a validated GlyT2 structural model including all ligand sites constructed by homology with the crystallized dopamine transporter from *Drosophila melanogaster*. Molecular dynamics simulations and energy analysis of the complex and functional analysis of a series of point mutants permitted to determine the structural determinants of ALX1393 and ORG25543 discrimination by GlyT2. The ligands establish simultaneous contacts with residues present in transmembrane domains 1, 3, 6, and 8 and block the transporter in outward-facing conformation and hence inhibit glycine transport. In addition, differential interactions of ALX1393 with the cation bound at Na1 site and ORG25543 with TM10 define the differential sites of the inhibitors and explain some of their individual features. Structural information about the interactions with GlyT2 may provide useful tools for new drug discovery.

## Introduction

Pain sensation is transmitted by afferent fibers connecting the peripheral tissues to the central nervous system. Inhibitory glycinergic interneurons in the dorsal spinal cord regulate the transmission of pain signals to the brain by operating through glycine receptors (GlyR) containing the α3 subunit at spinal cord synapses.^1,2^ The reduction of glycinergic inhibitory transmission by application of the GlyR antagonist strychnine produces hyperalgesia,^3^ while the intrathecal application of glycine prevents it.^4^ Thus, the mechanisms that allow increasing glycine levels in the spinal cord synapses of the dorsal horn can produce analgesia.^5^

Released glycine is cleared from the synaptic cleft by two plasma membrane glycine transporters GlyT1 and GlyT2.^6^ GlyT1 cotransports glycine, 2 Na^+^ ions, and 1 Cl^–^ ion into glial cells, exerting key control over extracellular glycine concentrations at glycinergic and glutamatergic pathways. GlyT2, exclusively expressed in presynaptic glycinergic neurons, takes up glycine together with 3 Na^+^ ions and 1 Cl^–^ ion and generates steeper glycine concentration gradients.^7,8^ GlyT2 supplies neurotransmitter to the presynaptic terminal for synaptic vesicle refilling, facilitating synaptic glycine recycling.^9^ Deletion of the GlyT2 gene in mice^10^ and loss-of-function mutations in the human GlyT2 gene, *SLC6A5*, abolish glycinergic neurotransmission. In humans, this causes a rare sensorimotor disorder called hyperekplexia or startle disease.^11,12^ Trivial touch or sounds trigger exaggerated startle responses and hypertonia in hyperekplexia patients, which can produce apnea episodes in newborns and even sudden infant death.^11,13,14^ Therefore, the total absence of GlyT2 function is pathological. In addition, complete inhibition of GlyT2 results in a severe facilitation of pain sensation most likely due to a complete breakdown in glycinergic inhibition in the dorsal horn. However, the local inhibition of GlyT2 can raise the extracellular glycine levels and enhance glycinergic neurotransmission in the dorsal spinal cord, which effectively suppresses pain transmission.^1^ GlyT2 is, thus, a potential target for controlling chronic pain states in humans, and the design of GlyT2 inhibitors is currently considered a challenging endeavor.^15^

Two selective GlyT2 inhibitors *O*-[(2-benzyloxyphenyl-3-fluorophenyl)methyl]-l-serine (ALX1393) and *N*-[[1-(dimethylamino)cyclopentyl]methyl]-3,5-dimethoxy-4-(phenylmethoxy)benzamide (ORG25543) with nanomolar affinity for the transporter have analgesic effects in pain animal models.^16^ ALX1393 has an antinociceptive effect on thermal, mechanical, and chemical stimulations in a rat acute pain model^17^ and also in several animal models of chronic pain.^15−17^ Although minimal side effects have been reported on locomotor activity in these reports,^18,19^ one study showed ALX1393 alleviates the bladder hypersensitive disorder but elicited significant increase in intercontraction interval and micturition pressure threshold.^21^ On the other hand, the pharmacokinetics properties of ALX1393 are improvable since it has been reported that only 5% of the drug crosses the blood–brain barrier.^18^ Moreover, ALX1393 is not fully selective for GlyT2, and it inhibits GlyT1 at concentrations above 0.5–1 μM in neuronal cultures and brain preparations.^15,19,20,22^ Nevertheless, a valuable feature of ALX1393 is its reversibility, an aspect that may minimize motor and respiratory side effects due to a low target residence time or fast dissociation kinetics.

ORG25543 also reduces allodynia in different pain models such as nerve ligation injury, streptozotocin-induced diabetic pain model, and complete Freund’s adjuvant-induced inflammatory pain.^19^ ORG25543 is more selective than ALX1393 for GlyT2 over GlyT1, and it seems to be an irreversible GlyT2 inhibitor.^18^ ORG25543 generates toxicity in animals due to its physiological irreversibility by affecting glycinergic neurotransmission in mouse spinal cord slices.^23^ It initially prolongs the glycinergic synaptic transmission, but at longer times, it reduces glycine neurotransmission by completely blocking GlyT2. If ALX1393 and ORG25543 display a competitive behavior, they may allow modulating GlyT2 inhibition by synaptic glycine concentrations.^24^ Overall, these inhibitors may have interesting properties as parental compounds for the development of more specific reversible GlyT2 inhibitors with short residence times at their binding sites and with the ability to pass the blood–brain barrier, which could be applied in pain therapy. Structural information about GlyT2 drug interactions is important for understanding their molecular mechanisms of action and will provide useful tools for new drug discovery.

GlyTs belong to the SLC6 family of neurotransmitter:sodium symporters (NSS), which includes the GABA and the monoamine transporters.^25^ The family encompasses membrane proteins with 12 transmembrane domains (TM) arranged in two topologically inverted structural repeats of 5 TMs each (TM1–5 and TM6–10). The repeats intertwine to form two bundles: a scaffold bundle including TMs 3, 4, 8, and 9 and a core bundle comprising TMs 1, 2, 6, and 7. The transport mechanism involves the rocking of the core bundle relative to the more rigid scaffold bundle to make accessible the central binding pocket alternatively to one side of the membrane or the other (alternating access).^25^ During the translocation cycle, the transporters undergo conformational changes that drive the protein to at least three conformational states: outward-open, occluded, and inward-open. The three of them have been crystallized in the prokaryotic homologue LeuT_Aa_ in the presence of substrates or inhibitors.^25−29^ Later, the eukaryotic *Drosophila melanogaster* dopamine transporter (*d*DAT)^30−32^ and the human serotonin transporter (*h*SERT)^33−36^ have been crystallized in inhibitor-bound forms.

Through molecular dynamics (MD) simulations on a validated GlyT2 structure modeled using LeuT_Aa_ as a template, we predicted the conservation of the substrate and two sodium binding sites.^37^ We, additionally, identified a transient sodium site in GlyT2 external vestibule, which confers isoform-specific properties to the transporter.^38^ Lately, a new refined GlyT2 model constructed by homology with the *d*DAT crystal^32^ permitted us to experimentally validate the location of the third sodium site in GlyT2 whose position remained obscure.^8^ We also verified that the region surrounding Na3 site has robust allosteric properties involved in the sensitivity to different cations. A model based in DAT also permitted others the analysis of the molecular determinants for substrate interactions at the glycine-binding site.^39^ Following preliminary experimental data of the inhibitor potency on different GlyT2 mutants, we profited the availability of our experimentally verified new GlyT2 modeled structure based on *d*DAT, which includes all the substrate-binding sites (Gly, 3 Na^+^ ions, and 1 Cl^–^ ion),^8^ and we carried out the molecular docking of ALX1393 and ORG25543. We performed MD simulations and energy analysis of the docking complexes and systematic functional assays of a series of point mutants that allowed us to predict some amino acids involved in the binding. Our approach revealed structural determinants for the selective inhibitors ALX1393 and ORG25543.

## Results and Discussion

### ALX1393 and ORG25543 Are Potent and Selective Noncompetitive GlyT2 Inhibitors

**Figure 1:**
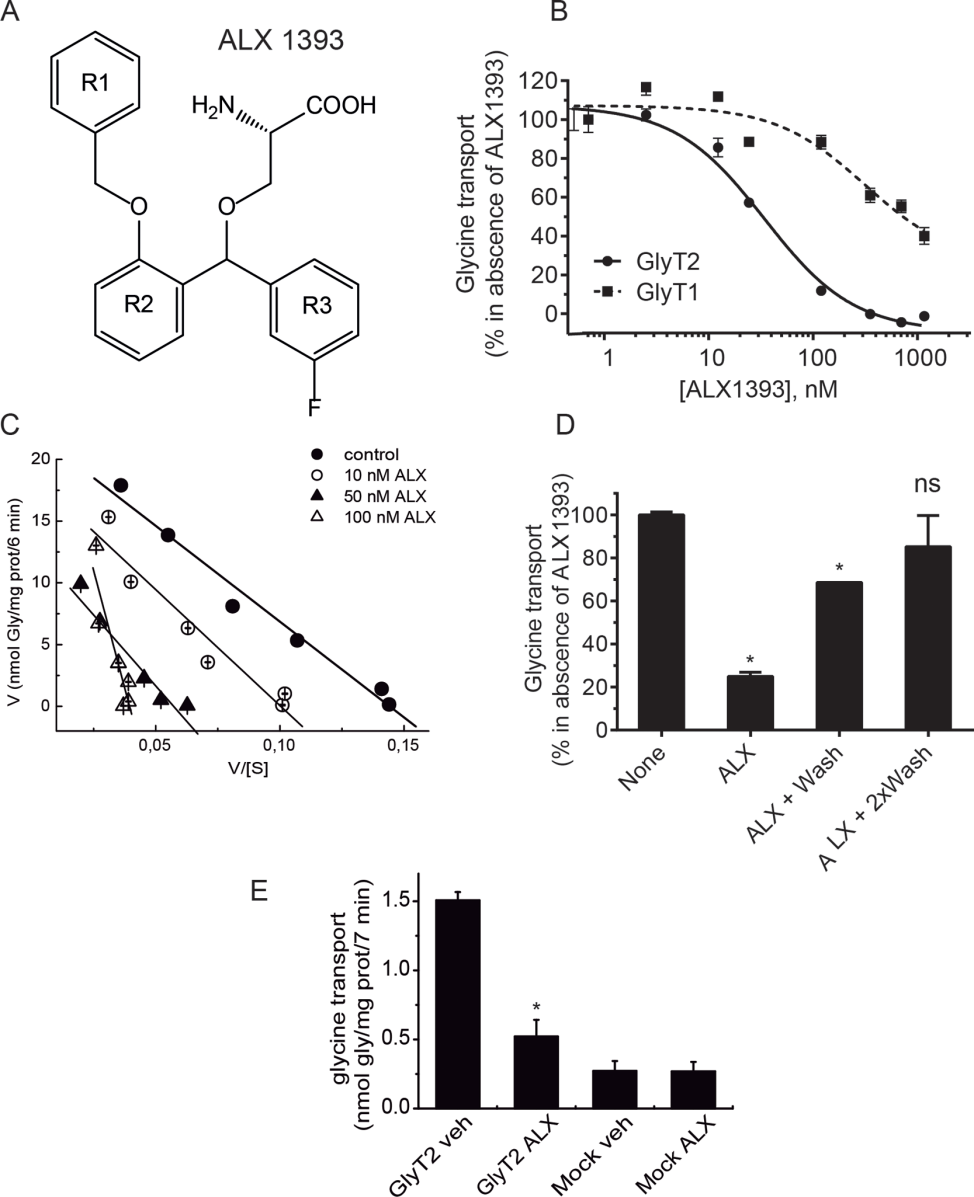
Characterization of GlyT2 inhibition by ALX1393. (A) Chemical structure of ALX1393 showing the four reference groups: amino acid and the three aromatic rings named R1, R2, and R3. (B) Concentration-dependent curves for GlyT1 and GlyT2. COS7 cells expressing the specified transporters were incubated in PBS with the indicated concentrations of ALX1393 or vehicle (B, C) and then subjected to [^3^H]glycine transport at 10 μM glycine (B, D) or increasing glycine concentrations (C), in the absence or presence of the same concentration of ALX1393 (B, C). (D) Cells expressing GlyT2 were incubated with PBS with 50 nM ALX1393 or vehicle (none), washed for the indicated times, and then assayed for glycine transport at 10 μM glycine in the absence of ALX1393. (E) Glycine transport inhibition by GlyT2 transfected and untransfected (mock) cells by 50 nM ALX1393 or vehicle. 100% glycine transport by GlyT2 and GlyT1 was: 1.5 ± 0.2 and 4.2 ± 0.3 nmol glycine/mg protein/7 min, respectively. *p < 0.05, ANOVA with Bonferroni′s posthoc test.

Figure 1 shows that ALX1393 inhibits the glycine transport by recombinant GlyT2 expressed in COS7 cells with an IC_50_ = 31 ± 2.7 nM and displays about 2 orders of magnitude selectivity for GlyT2 over GlyT1 (IC_50_ in the low μM range). This is in agreement with previous reports of an IC_50_ for GlyT2 in the nM range and with the reported selectivity of the compound in heterologous and brain-derived experimental systems.^15,19^ The chemical structure of ALX1393 is shown in Figure 1A. This compound (*O*-[(2-benzyloxyphenyl-3-fluorophenyl)methyl]-l-serine) contains an amino acid core, derivative of l-serine with α-amino, and carboxyl moieties and a three aromatic ring groups O-substituted in the hydroxyl side chain. For the ease of description, the three aromatic rings in the structure have been named R1 (benzyl ring), R2 (oxyphenyl ring), and R3 (fluorophenyl ring). According to the presence of the amino acid moieties, ALX1393 is predicted to exert a competitive inhibition of GlyT2 function. In order to establish the inhibition type, we measured glycine transport sensitivity to three concentrations of the inhibitor that span at least an order of magnitude using a wide range of glycine concentrations (0.5–500 μM), and we obtained Eadie–Hofstee plots (Figure 1C). The plots mostly indicate a noncompetitive behavior, although at higher inhibitor concentrations, ALX1393 may act competitively (measured *K*_m_s at 1, 10, 50, and 100 nM ALX1393 were: 155.2, 188.0, 230.0, and 760.4 μM and the *V*_max_s were 22.3, 18.8, 13.2, and 30.4 nmol Gly/mg prot/6 min, *n* = 3). The EC_50_ for ALX1393 at every glycine concentration differed in no more than 30% of its value at 10 μM glycine. In addition, the inhibition could be eliminated by cell washing, confirming the reversibility of the inhibition (Figure 1D).

**Figure 2:**
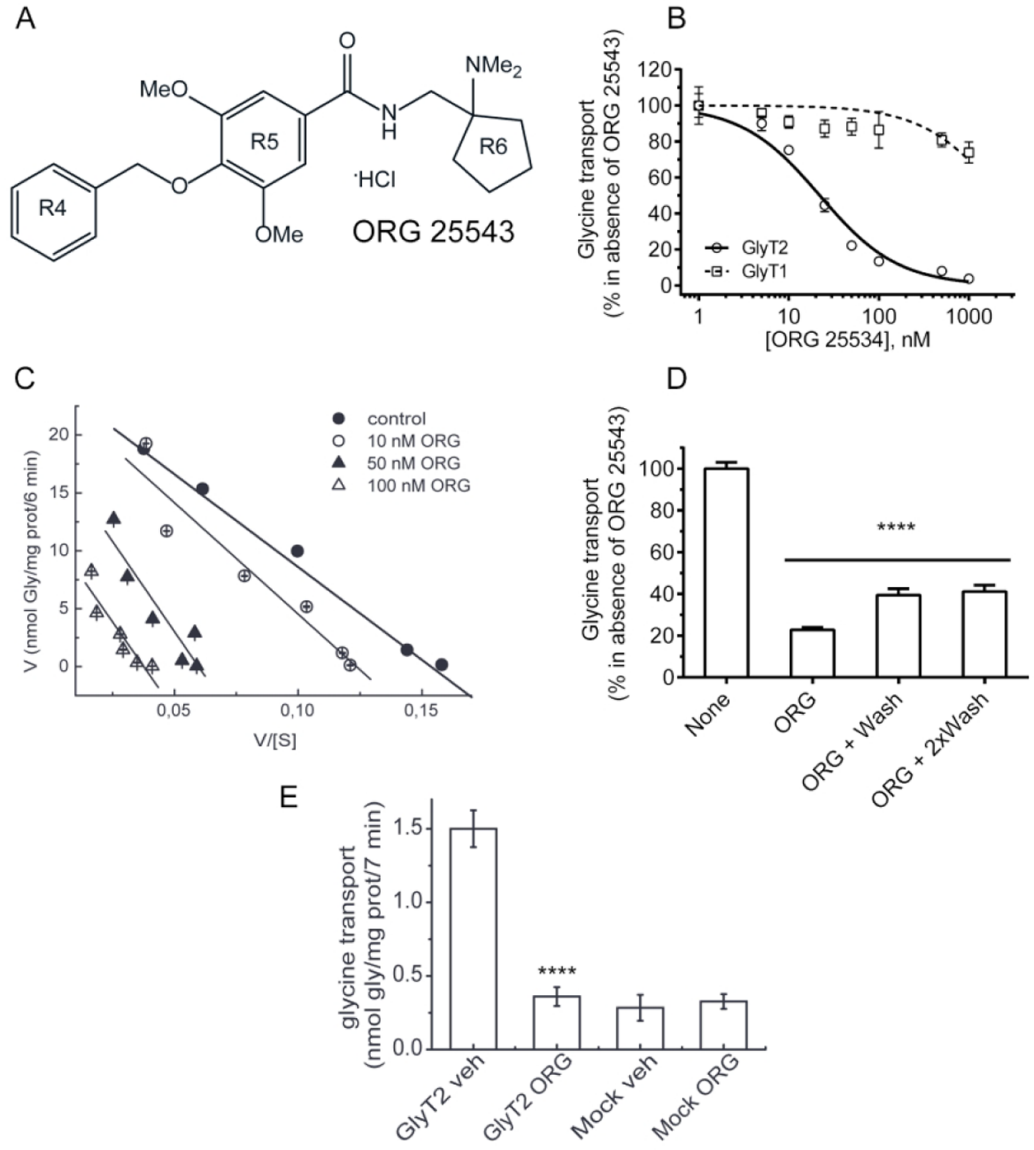
Characterization of GlyT2 inhibition by ORG25543. (A) Chemical structure of ORG25543 showing the four reference groups: amino acid and the three aromatic rings named R4, R5, and R6. (B) Concentration-dependent curves for GlyT1 and GlyT2. COS7 cells expressing the specified transporters were incubated in PBS with the indicated concentrations of ORG25543 or vehicle (B, C) and then subjected to [^3^H]glycine transport at 10 μM glycine (B, D) or increasing glycine concentrations (C), in the absence or presence of the same concentration of ORG25543 (B, C). (D) Cells expressing GlyT2 were incubated with PBS with 50 nM ORG25543 or vehicle (none), washed for the indicated times, and then assayed for glycine transport at 10 μM glycine in the absence of ORG25543. (E) Glycine transport inhibition by GlyT2 transfected and untransfected (mock) cells by 50 nM ORG25543 or vehicle. 100% glycine transport by GlyT2 and GlyT1 was: 1.5 ± 0.2 and 4.2 ± 0.3 nmol glycine/mg protein/7 min, respectively. ****p < 0.0001, ANOVA with Bonferroni′s posthoc test.

ORG25543 is another selective GlyT2 inhibitor, with an IC_50_ also in the nM range (17.7 ± 4.6 nM, Figure 2B), in agreement with previous reports.^18^ The compound (*N*-[[1-(dimethylamino)cyclopentyl]methyl]-3,5-dimethoxy-4-(phenylmethoxy)benzamide) also contains three rings that were named R4 (benzyl ring), R5 (phenylmethoxy ring), and R6 (cyclopentyl ring) (Figure 2A), and it is an inhibitor with higher selectivity for GlyT2 (Figure 2B). The Eadie–Hofstee plots obtained at three concentrations of the inhibitor that span at least an order of magnitude using a wide range of glycine concentrations (0.5–500 μM) indicate a mainly noncompetitive behavior (measured *K*_m_s at 1, 10, 50, and 100 nM ORG25543 were: 160.3, 192.4, 311.3, and 306.3 μM, and the *V*_max_ were 24.6, 23.8, 18.6, and 11.5 nmol Gly/mg prot/6 min, *n* = 3). The EC_50_ for ORG25543 at every glycine concentration was slightly different, reaching values that even doubled its value at 10 μM glycine (Figure 2C). Unlike ALX1393, ORG25543 can cross the blood–brain barrier, a pharmacokinetic property that makes this GlyT2 inhibitor a potential therapeutic candidate for pain treatment.^18^ However, the proposed irreversible inhibition of GlyT2 by ORG25543 (Figure 2D) makes ORG25543 a hazardous tool for *in vivo* treatment of pain, since it may generate toxicity and side effects. Taking into account that reversible inhibition is a valuable pharmacokinetic property, we wished to elucidate the molecular reasons for the reversibility of ALX1393 binding to GlyT2 by comparing ALX1393 and ORG25543 binding mode to the transporter using molecular docking on a GlyT2 modeled structure based on *d*DAT.^8^

### Comparative Molecular Docking of ALX1393 and ORG25543

**Figure 3:**
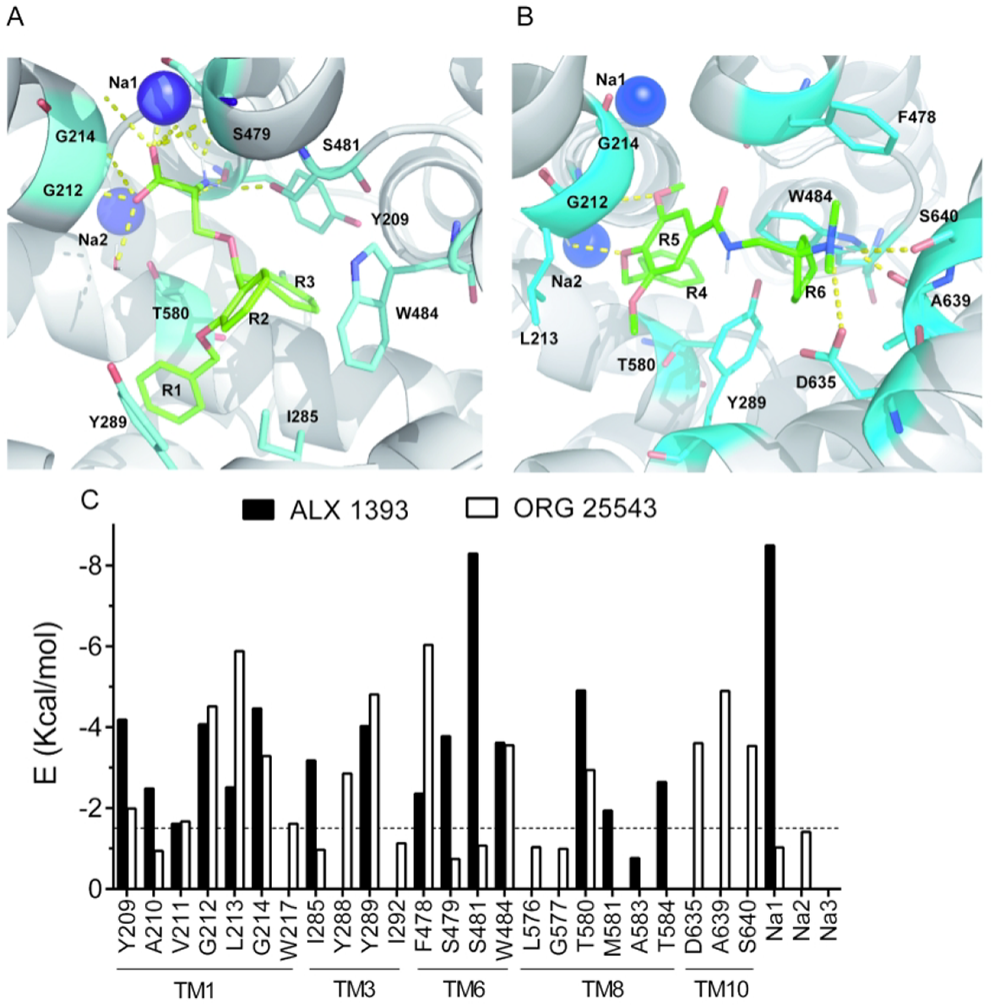
Final poses of GlyT2 bound to the inhibitors. (A) GlyT2-ALX1393 complex. (B) GlyT2-ORG25543 complex. Transporter is shown from the extracellular side in cartoon mode gray, and the relevant interactions are shown as cyan sticks colored according to the atoms: red, blue, and gray for oxygen, nitrogen, and hydrogen, respectively. Cations are shown as purple spheres. Ligands are represented as green sticks colored as above. (C) Interaction energies of the predicted contacts of GlyT2 with ALX1393 and ORG25543 as a result of the docking and MD simulations. Dash line represents energy values of 1.5 kcal/mol.

Preliminary experimental analysis based on the potency of the inhibitors on different GlyT2 mutants suggested several candidate residues as molecular determinants for inhibitor interactions. To validate our predictions, we performed the molecular docking of ALX1393 or ORG25543 to the GlyT2 model.^8^ We chose placing the docking box in the transporter pore for the two ligands because we wanted to know whether the binding site was common or different for the two inhibitors. We used two independent computational docking methodologies of reasonable flexibility of ligand and protein to compare the interactions of the two inhibitors within the same system: Autodock Vina and Glide (Schrödinger). ALX1393 and ORG25543 were placed at a coordinate in the GlyT2 model equivalent to the substrate-binding site in *d*DAT and allowed to randomly translate. Among the several docking poses obtained from the interaction between the ligand and the target protein, the ones exhibiting the best docking score, which estimates the strength of the interaction, were chosen. In addition, we compared the ligand–protein interactions of the obtained ligand-docked poses with the interactions shown by *d*DAT crystals bound to the tricyclic antidepressants (TCA) nortryptilin (PDB: 4M48)^32^ and reboxetine (PDB: 4XNX).^33^Figure 3A,B shows the binding modes adopted by the two inhibitors ALX1393 and ORG25543 in its interaction with the GlyT2 model. Interestingly, the best score poses were the ones closest to those described for the interaction of ligands with *d*DAT or hSERT structures.^30,31,34^ The inhibitors were anchored to the bottom of the transporter channel, by important van der Waals and electrostatic interactions including multiple hydrogen bonds. The ligands established simultaneous contacts with residues present in TMs 1, 3, 6, and 8 and, in the case of ALX1393, with the cation bound at Na1 site. Instead, ORG25543 interacts with the TM10 in addition to TMs 1, 3, 6, and 8. Since these residues were located in both the core (TM1, 2, 6, and 7 and Na1 site) and the scaffold (TM3, 4, 8, and 9) bundles,^40,41,42^ the binding of any of the two inhibitors may block the rocking of the core bundle relative to the scaffold and hence inhibit glycine transport.

Table 1 shows the main residues of GlyT2 interacting with ALX1393 and the type of interaction. The positively charged nitrogen atom of the α-amino moiety of ALX1393 forms hydrogen bonds with the carbonyl backbones of Tyr-209 and with lower energy with those of Ser-479 and Ala-210 (Figure 3A,C and Table 1). Tyr-209 is the equivalent residue to *d*DAT Phe-76, whose carbonyl group of the main chain forms a hydrogen bond with the amine group of the *d*DAT-bound antidepressant ligand.^30,31^ In SERT, a residue equivalent to Tyr-209 (Tyr-95) is also necessary for interactions with bound antidepressants.^33,38,39^ Besides, the ligand amino group interacts with the side chain of Ser-481 through hydrogen. The carboxyl group of ALX1393 establishes electrostatic interactions with the backbone of residues forming the glycine substrate site (Gly-212, Gly-214) and, importantly, with the sodium ion located in Na1 site. In a similar way, TCAs coordinate with the sodium ion in the Na1 site through a water molecule. However, the interactions predicted to have a key role in the stabilization of the pose are those affecting the ligand aromatic rings. ALX1393 rings were locked by van der Waals interactions with aliphatic chains and π–π type stacking with aromatic side chains of the transporter. Main interactions occurred with the side chains of Ser-481, Thr-580, Tyr-289, Trp-484, Ile-285, Thr-584, and Phe-478. It is worth noting that three of the above residues interact with more than one ligand ring: Thr-580 (R1 and R3), Tyr-289 (R1 and R2), and Trp-484 (R2 and R3).

**Table 1:** Main Residues of GlyT2 Interacting with ALX1393 and Type of Interaction^a^

GlyT2 residue	location (TM)	energy (kcal/mol)	interaction type	ALX1393 moiety
Na1	TM1,6,7	–8.47	q–q	–COO^–^
S481	TM6	–8.32	Hb + vdW	–NH_3_^+^/–R3
T580	TM8	–4.97	vdW	–R1/–R3
G214 (BB)	TM1	–4.49	Hb	–COO^–^
Y209 (BB)	TM1	–4.22	π–π stacking + Hb	–R3/–NH_3_^+^
G212 (BB)	TM1	–4.09	Hb + vdW	–COO^–^
Y289	TM3	–4.05	π–π stacking	–R1/–R2
S479 (BB)	TM6	–3.79	Hb	–NH_3_^+^
W484	TM6	–3.66	π–π stacking	–R2/–R3
I285	TM3	–3.20	vdW	–R2
T584	TM8	–2.70	vdW	–R1
L213 (BB)	TM1	–2.53	vdW	–COO^–^/–R1
A210 (BB)	TM1	–2.45	Hb	–NH_3_^+^
F478	TM6	–2.35	π–π stacking	–R2

a vdW: van der Waals; Hb, hydrogen bond; q–q, charge–charge; BB, backbone.

ORG25543 is bound to the transporter by multiple van der Waals interactions and hydrogen bonds (Figure 3B,C and Table 2). The main interacting residue is Phe-478 whose side chain stabilizes R6 and the *N*-dimethylamino group by van der Waals contacts. This moiety also interacts with the backbone of Ala-639 and the side chains of two other residues in TM10: Asp-635 and Ser-640 through hydrogen bonding and electrostatic interactions. It is worth noting that Asp-635 is involved in the formation of the extracellular gate through a salt bridge with Arg-218. The interactions involving TM10 residues are unique features of ORG25543, not found in the interaction of ALX1393 with GlyT2 or in additional inhibitors of other members of the SLC6 family of transporters. The side chain of Ala-639 additionally holds the R6 through van der Waals interactions. Furthermore, the moieties substituting R4 interact with TM1 residues (Gly-212 and the backbone of Leu-213 and Gly-214). ORG25543 rings are held by the side chains of Tyr-289 (R4 and R5), Trp-484 (R4, R5, and R6), Thr-580 (R4), and Tyr-288 (R6). A comparative view of the main GlyT2 residues predicted to be involved in ALX1393 and ORG25543 coordination is shown on Figure 3C.

**Table 2:** Main Residues of GlyT2 Interacting with ORG25543 and Type of Interaction^a^

GlyT2 residue	location (TM)	energy (kcal/mol)	interaction type	ORG25543 moiety
F478	TM6	–6.36	vdW	–NMe_2_/–R6
L213 (BB)	TM1	–5.83	Hb + vdW	–OMe/–O-
A639 (BB)	TM10	–5.41	Hb + vdW	–NMe_2_/–R6
Y289	TM3	–5.01	π–π stacking	–R4/–R5
D635	TM10	–4.61	q–q + Hb	–NMe_2_
G212	TM1	–4.52	vdW	–OMe
S640	TM10	–4.33	Hb	–NMe_2_
W484	TM6	–3.72	π–π stacking + vdW	–R4/–R5/–R6
G214 (BB)	TM1	–3.39	Hb	–OMe
T580	TM3	–2.93	vdW	–R4
Y288	TM8	–2.91	VdW	–R6

a vdW: van der Waals; Hb, hydrogen bond; q–q, charge–charge; BB, backbone.

### Structural and Energetic Stability of the Poses

The best fit docking poses obtained were further refined by 50 ns MD simulations performed with AMBER to ascertain whether the complexes were structurally and energetically stable over time. To assess convergence, the root-mean-square deviation (RMSD) values of the inhibitor-GlyT2 complexes were calculated over the simulation time (Supplementary Figure S1A,B). Global RMSD values of the transporter were lower than 2 Å, as expected for a stable protein model. In addition, Na1, Na2, Na3, and Cl^–^ ions were very stable along the dynamics with RMSD values lower than 1.5 Å. Regarding the ligands, after the first 2 ns of simulation corresponding to the equilibration step, they changed the pose with respect to the docked structure, reaching values of 3.5 Å RMSD and everyone reached its binding site. The new pose was stable, and no changes were observed in RMSD values along the MD, which means that during the rest of the simulation, the ligands remained at their position. The potential fluctuations in the ligand binding region were also monitored by the shown root-mean-square fluctuation (RMSF) values. As expected for a membrane protein surrounded by lipids, the main structural movements corresponded to regions in contact with the solvent such as terminal ends and TM-connecting loops. Supplementary Figure S2A,B shows that, besides the terminal ends which are charged regions with greater freedom of movement, the main fluctuations were observed in the extracellular loops EL2 (TM3 and 4, residues 311–351), protein regions that are expected to respond to substrate binding. However, besides EL2, none of the rest of the RMSF values exceeded 3 Å. In addition, there were no remarkable fluctuations affecting the ligand binding site. Furthermore, protein–ligand interaction energies measured during the simulations remained nearly constant, indicating the poses of the ligands in the protein were energetically favorable (Supplementary Figure S3A,B). Although we obtained high interaction energy values (around −65/–70 kcal/mol), probably due to the fact that the system does not include all the conformational changes that are coordinately required for binding, the values were comparable for the two ligands. In order to know whether these interactions underwent time-dependent changes during the MD, we analyzed the evolution of the interaction energy of every key residue during the simulation. The obtained results are shown in Figure S4A and indicate that the major interactions of the ligand with the protein were stable over time.

### Experimental Validation of the Molecular Interactions

To validate the predicted interactions between the inhibitors and GlyT2, we used systematic site-directed mutagenesis to construct substitution mutants of the potential residues involved in ligand binding. The mutants were expressed in COS7 cells, and the inhibitory action of ALX1393 or ORG25543 on the glycine transport by the mutants was assessed. The GlyT2 residues predicted to interact with the inhibitors through its side chain were replaced by amino acids expected to force the loss of the interaction. In the cases where the interaction occurred through the backbone, the inserted amino acid sought to alter the disposition of the substituted residue to lose the coordination. Supplementary Table S1 shows some of the features of the analyzed mutants. Most of the mutants were active, although depicted lower activity as compared to the wild-type. However, many residues involved in the docking are also involved in the binding of substrate(s) or in the stabilization of the transporter structure, and for this reason, some of the mutants were inactive. For example, the different substitutions made in the Tyr-289 and Tyr-209 resulted in inactive transporters. These two residues are preserved throughout the NSS family, and Tyr-289 has proven to have an important structural role in stabilizing the unwound portion of the TM1 helix, whereas Tyr-209 has a key role in S1 site formation through its interaction with Ser-481.^25,30,33^ Other residues such as Leu-213 or Ser-479 are involved in the formation of the glycine-binding site and Na^+^-binding site, respectively. So, their substitutions yielded inactive transporters as well.

**Figure 4:**
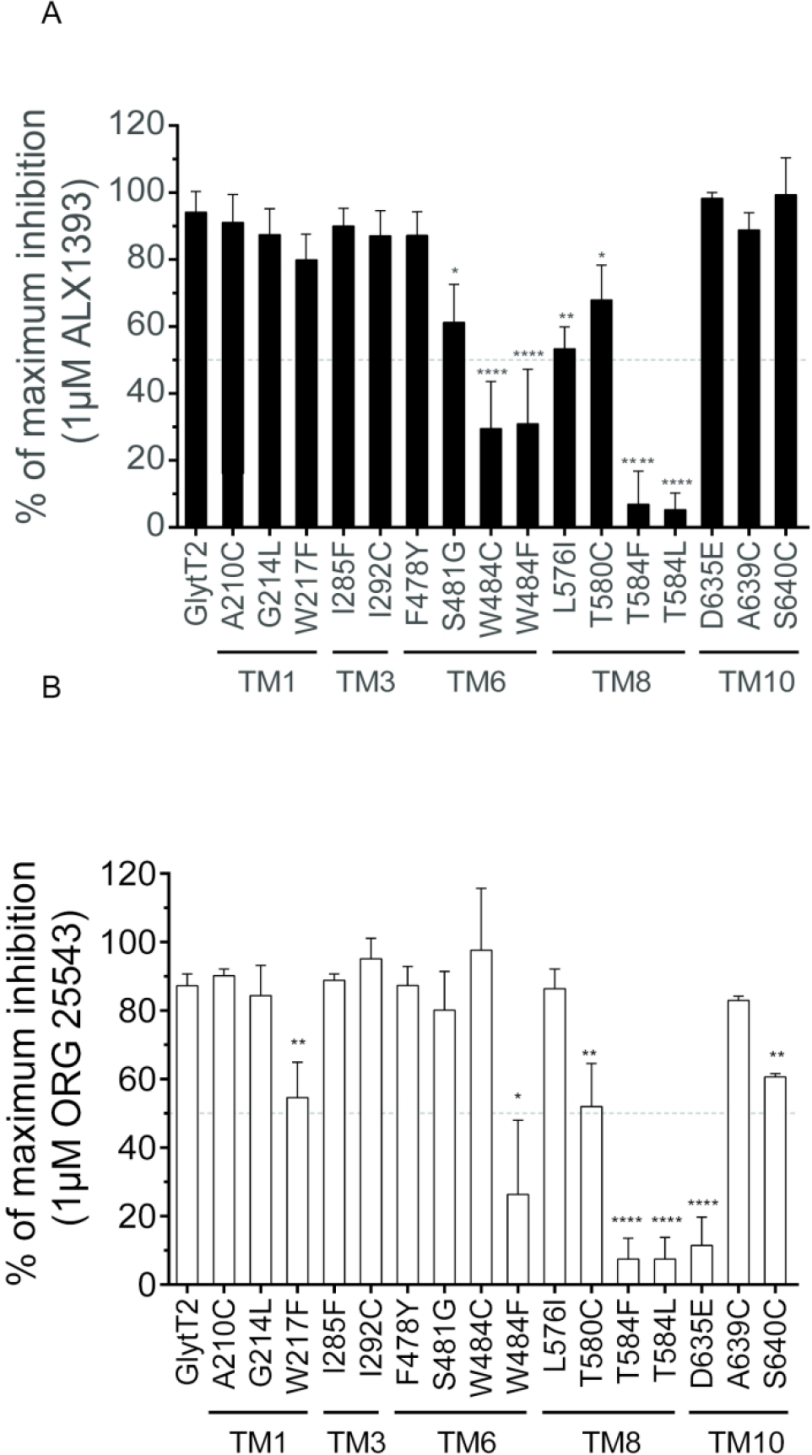
Experimental validation of GlyT2-inhibitor molecular interactions. Sensitivity to ALX1393 (A) and ORG25543 (B) of GlyT2 mutants. Glycine transport by COS7 cells expressing GlyT2 treated for 10 min with vehicle or 1 μM of ALX1393 (A) or 1 μM of ORG25543 (B) in the presence of the μM glycine concentrations according their EC_50_: GlyT2, D635C, A639C, and S640C (10 μM); G214L (5 μM); I292C and T584F (15 μM); W217F (20 μM); A210C, F478Y, S481G, and L576I (25 μM); I285F and T580C (50 μM); W484F (75 μM); T584L (120 μM); and W484C (200 μM). The histogram bars represent the reached percentage of the maximum inhibition of the transport activity. Error bars are SEM. Significantly different from GlyT2: *p < 0.05, **p < 0.01, ***p < 0.001, ****p < 0.0001 in ANOVA with Bonferroni’s multiple comparison test.

Figure 4 shows the maximum inhibition of [^3^H]-glycine transport by the relevant active mutants in the presence of fully inhibitory concentrations (1 μM) of ALX1393 or ORG25543. Some of the mutations did not interfere with the inhibitory potency of the inhibitors. These included mutations on residues predicted to bind the ligand with the backbone (A210C, G214L, and A639C), mutations where the substitution failed to prevent the binding (I285F and F478Y), and mutations on residues predicted to have a nonsignificant interaction with the ligand (I292C). T580C mutation impaired the inhibitory action of both compounds to a similar extent since this residue has a key role in the S1 glycine-binding site.^8^ Our experimental evidence pointed to some common residues important for the binding of both ALX1393 and ORG2554. Those are Thr-580 and Thr-584 to which we probably have to add some of the residues that yielded inactive transporters upon nonconservative substitution such as Tyr-289, Phe-478, and Ser-479, involved in the binding of substrate(s) or in the stabilization of the active center of the transporter.^43^ Regarding Thr-584 mutations, the insertion of the voluminous Leu or Phe was not tolerated by the two ligands, although Thr-584 does not directly bind ORG25543. The natural nonconservative substitution present in GlyT1 (T584L, constructed in GlyT2) showed a reduced inhibition potency for both ligands. However, the other nonconservative substitution S481G showed more reduced inhibition potency for ALX1393 than for ORG25543, according to the data in Tables 1 and 2. Also, Trp-484 mutants were sensitive to both inhibitors, although the effectivity of the substitution was different for the two inhibitors (see below). Phe-478 is also not conserved in GlyT1, but it is replaced by a tyrosine through a conservative substitution (F478Y) that functionally replaces the original amino acid. We made some less conservative substitutions, but replacements of Phe-478 to cysteine, alanine, and methionine were not functional (not shown), preventing us from reaching a solid conclusion about the specificity of this position.

On the other hand, there are mutations that exerted differential effects on the inhibitory potency of ALX1393 and ORG25543 (W217F, W484C, L576I, S481G, D635E, and S640C), and these effects are in agreement with the docking predictions. The substitution of Trp-217, which is one helix turn above Leu-213, selectively reduced ORG25543 inhibition according to the stronger binding energy measured as compared to ALX1393. Mutations on Trp-484 probably interfere with the access and accommodation of both ligands when substituted by Phe, but only with ALX1393 when Trp-484 is substituted by Cys. This agrees with a position for ALX1393 located below within the channel as compared to ORG25543. Nonetheless, the most interesting mutations affected residues predicted by the docking as exclusive for each ligand. In contrast to ORG25543, ALX1393 binds to the sodium bound in Na1. For this reason, mutant L576I, which disrupts the Na2 site^37^ and indirectly affects Na1, prevented the inhibition by ALX1393 to a greater extent than that of ORG25543. Finally, mutations in the two TM10 residues predicted to interact with their side chain with ORG25543, but not with ALX1393, selectively impaired ORG25543 inhibition.

**Figure 5:**
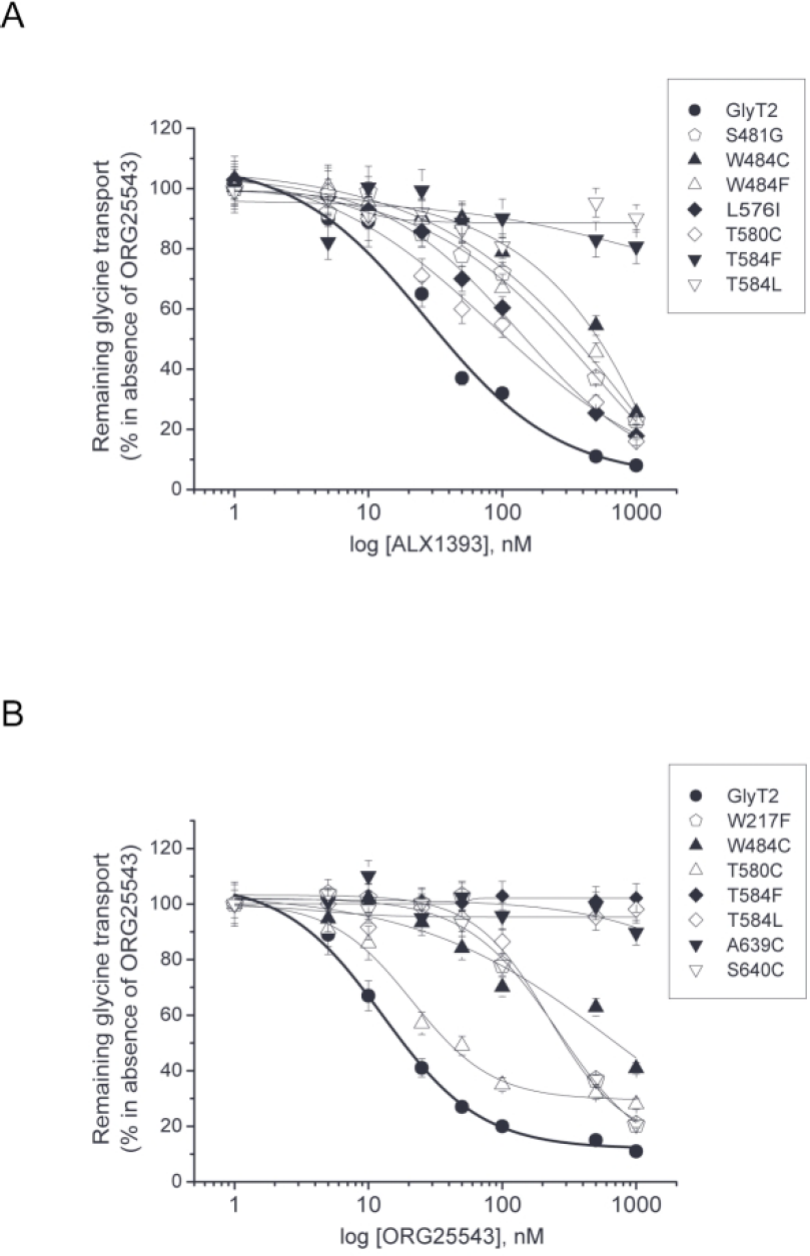
Concentration–response curves of glycine transport by partially resistant mutants. Sensitivity to ALX1393 (A) or ORG25543 (B) of the indicated GlyT2 mutants expressed in COS7 cells. Glycine transport by cells treated for 10 min with vehicle or the indicated concentrations of ALX1393 (A) or ORG25543 (B) in the conditions described in Figure 4.

In order to better quantify the alterations of the inhibitory potency of ALX1393 and ORG25543 on the relevant mutants, dose–response curves were performed at increasing inhibitor concentrations reaching high μM values (Figure 5). From these curves, the IC_50_ value was estimated for the different mutants (Supplementary Table S1). As expected, the determined IC_50_ was in good agreement with the magnitude of the inhibition at 1 μM shown in Figure 4. Mutations that did not alter the inhibitory potency (i.e., G214L, A210C, and F478Y) gave IC_50_ values comparable to wild-type. Conversely, the group of mutants with the lowest maximum inhibition at 1 μM compound showed resistance to inhibition and yielded IC_50_ values at least 10 times higher than that of GlyT2 wild type.

### Differential Interactions of ALX1393 and ORG25543

**Figure 6:**
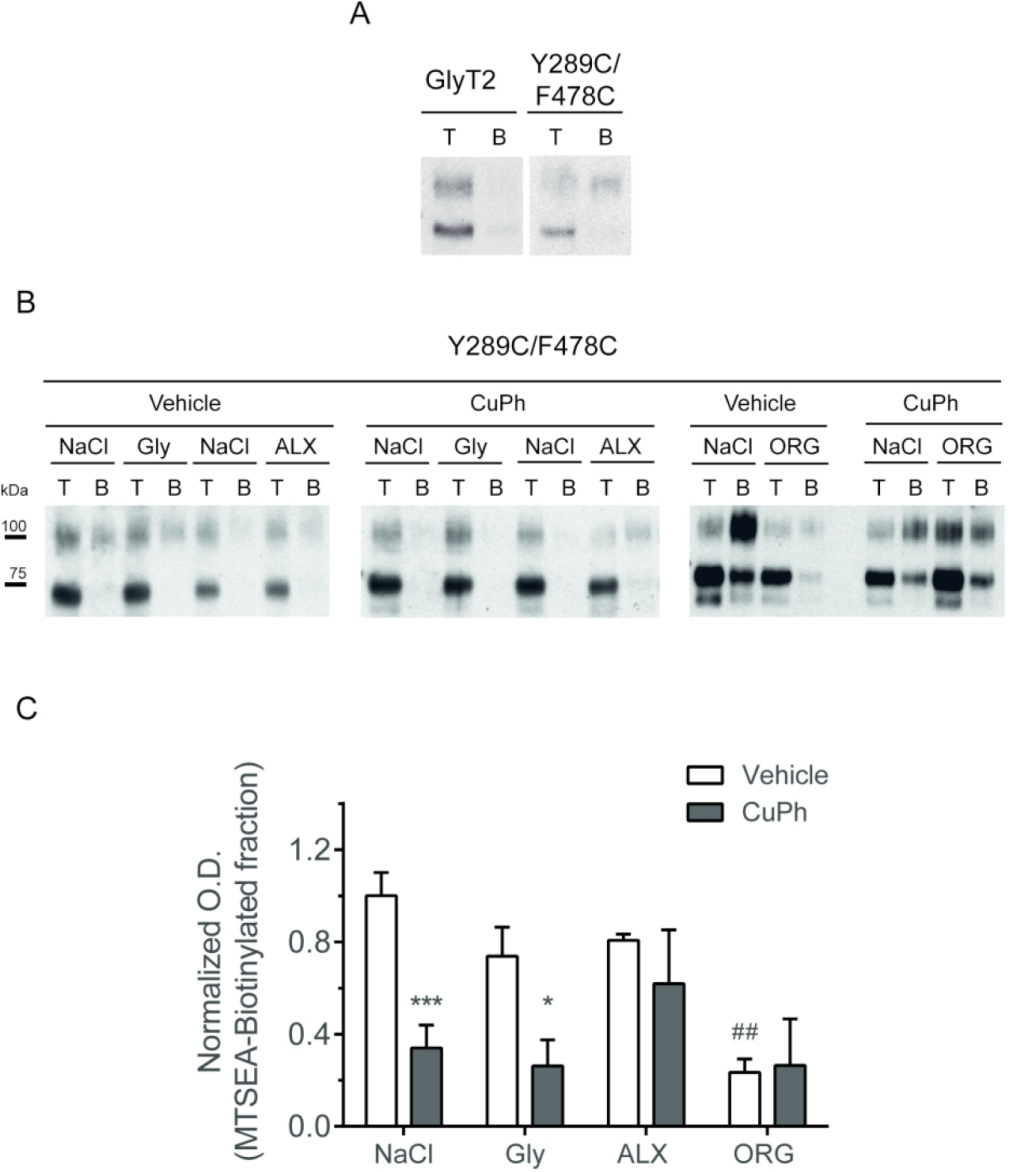
MTSEA-biotin labeling of the inner external gate double mutant Y289C/F478C. (A) COS7 cells expressing wild-type GlyT2 or the Y289C/F478C mutant of GlyT2 were subjected to MTSEA-biotinylation as described in the Methods section (T, total transporter; B, MTSEA-biotinylated transporter). (B) COS7 cells expressing the Y289C/F478C mutant of GlyT2 were preincubated with the irreversible oxidant reagent cupper-phenantroline (CuPh) before the MTSEA-biotin staining in PBS (NaCl) or PBS supplemented with the indicated additions: 1 mM glycine (Gly), 1 μM ALX1393 (ALX), or 1 μM ORG25543 (ORG). Then, the cells were washed and subjected to MTSEA-biotinylation as described above. Western blot for GlyT2 detection of a SDS-PAGE loaded with total proteins (lanes T) and biotinylated proteins (lanes B) at a ratio 1:10. (C) Densitometric analysis of the percentage of total transporter (B as a % of T) that was MTSEA-biotin labeled in each condition. Significantly different from vehicle: *p < 0.05, ***p < 0.001 in Student’s *t* test. Significantly different from NaCl: ##*p* < 0.01 in Student’s *t* test.

We next tried to substantiate some of the differential interactions of ALX1393 and ORG25543 with GlyT2 by using a transport-independent approach. Our first question was to analyze whether the bound inhibitors indeed maintain the transporter in the outward-open conformation, as hypothesized. We found that externally accessible cysteines^38^ introduced in TM10 (at positions A639 or S640) were protected from external MTSEA-biotin labeling by the inhibitors, although this approach was not sensitive enough to reach significance (not shown). Besides, the maximal protection was exerted in the presence of the substrates (glycine, Na^+^ and Cl^–^), a condition that promotes the transition of the transporter to the occluded conformation and masks the target cysteine.^45^ For this reason, we questioned the maintenance of the outward-open conformation during inhibitor binding and tried an alternative approach to answer this inquiry. We generated a double GlyT2 mutant (Y289C/F478C) by introducing two cysteines replacing the two amino acids that form the inner external gate. As depicted in Figure 6A, the MTSEA-biotin label of the plasma membrane double mutant was more intense than that of the wild type, indicating the mutant traffics properly to the plasma membrane and the reagent can get external access to the introduced cysteines. The two incorporated cysteines are predicted to be close enough to form a disulfide bond upon oxidation, and the formation of this bond would make the cysteines no longer accessible to the external −SH reagent. Therefore, we preincubated COS7 cells expressing the double mutant with the irreversible oxidant reagent cupper-phenantroline (CuPh) before the MTSEA-biotin labeling and monitored whether the binding of the inhibitors permittedthe cross-linking of the gate residues. As expected, in the absence of any inhibitor, the CuPh treatment reduced the transporter label indicating the closure of the external gate (Figure 6B,C). In contrast, the label was maintained in the presence of the inhibitors, indicating they do not permit the cross-linking of the cysteines, according to the maintenance of the outward-facing conformation. To confirm the results, we performed the same experiment in the presence of the substrates of GlyT2: glycine, Na^+^, and Cl^–^. As expected, in this condition, the label was lost, indicating the substrates permitted the closure of the external gate and thereby the transition to an occluded conformation. It is worth noting that the two cysteines introduced in the double mutant substituted two amino acids which were predicted to have a role in the binding of the two inhibitors, but more prominent in that of ORG25543 (Tables 1 and 2). For this reason, the labeling of the transporter mutant was reduced in basal conditions (vehicle), but especially in the presence of ORG25543 (Figure 6B,C). This fact obligated us to expose the western blot films to a higher extent to visualize the label of the 100 kDa surface protein. In this condition, some staining on the 75 kDa intracellular transporter with MTSEA-biotin became apparent. The labeling could be due to a partial penetration of the reagent or (most likely) to some unspecific staining by the extra reagent during the washes of the lysate that followed the 4 °C labeling of the cells. We carefully evaluated that this unspecific labeling on the 75 kDa bandwas an invariant percentage of the total transporter in all the situations. The 75 kDa band was more prone to be labeled, probably due to misfolding that exposes reactive cysteines. However, the 75 kDa label was minimal in the biotinylated samples except in the experiments where ORG25543 was involved, in which the higher exposures made the label on the 75 kDa band apparent even in the biotinylated samples.

Thus, the prediction suggesting the inhibitors lock the transporter in the outward-facing conformation was proven by protection experiments, as the inhibitors protect the transporter from the labeling at two cysteines introduced in substitution of the inner external gate residues (Y289C/F478C). This behavior could not be tested for the external gate residues R218/D635, since ORG25543 interacts with D635 by electrostatic interactions and its substitution by a cysteine prevents inhibitor binding and, therefore, protection (not shown).

### ALX1393 and ORG25543 Have Different Sodium Dependence

**Figure 7:**
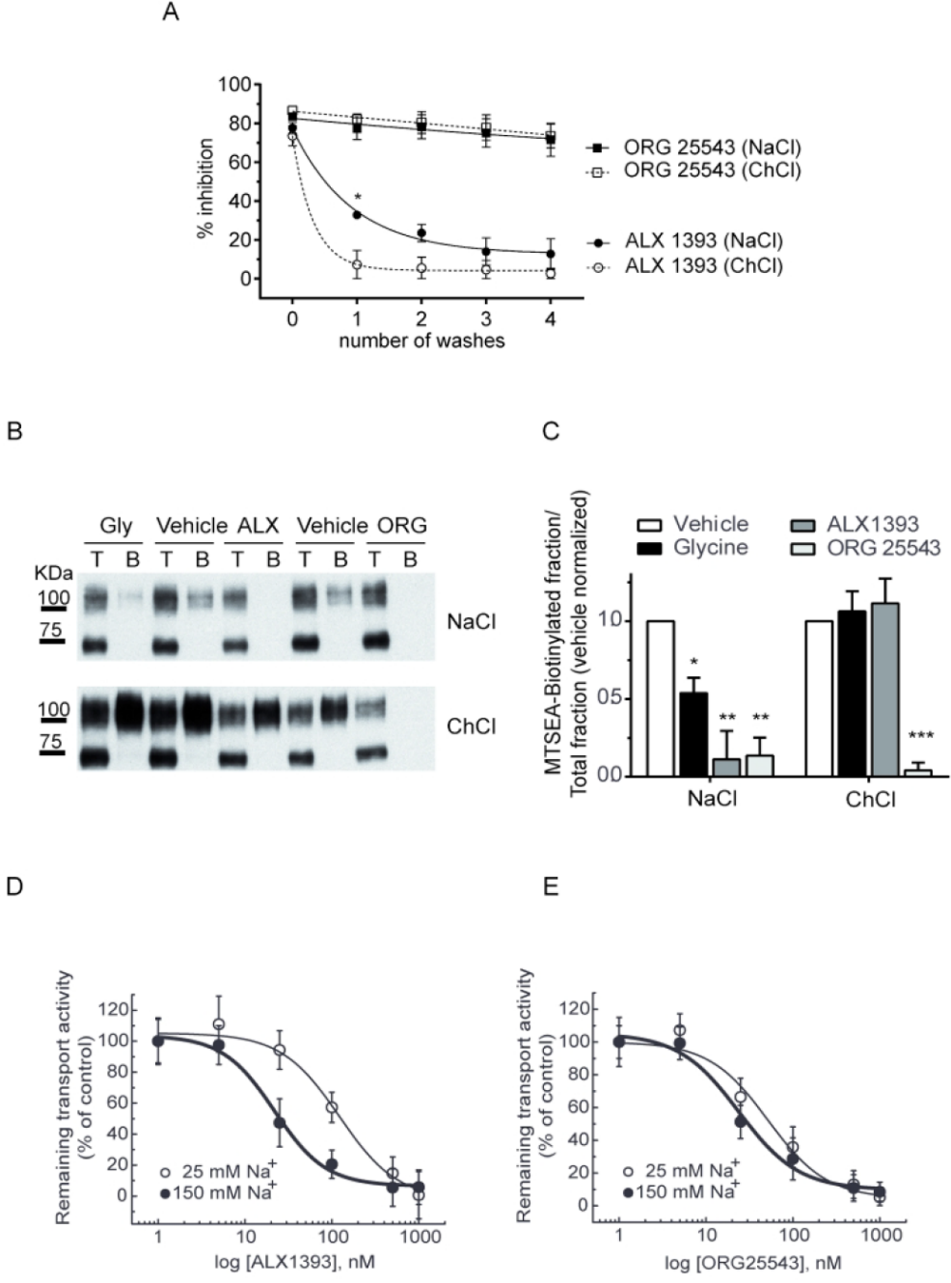
Sodium effects on ALX1393 and ORG25543 inhibition. (A) COS7 cells expressing GlyT2 were treated for 10 min with vehicle or 1 μM of ALX1393 or 1 μM of ORG25543 in HBS containing NaCl and then subjected to 1–4 washes with HBS containing NaCl or choline chloride (ChCl). [^3^H]-Glycine transport was measured after each wash and depicted as percentage of transport inhibition. (B) COS7 cells expressing the GlyT2 mutant A223C were subjected to MTSEA-biotinylation in HBS containing NaCl or ChCl in the presence of vehicle or 1 μM of ALX1393 or 1 μM of ORG25543. Western blot for GlyT2 detection of a SDS-PAGE loaded with total proteins (lanes T) and biotinylated proteins (lanes B) at a ratio 1:10. (C) Densitometric analysis of the percentage of total transporter (B as a % of T) that was MTSEA-biotin labeled in each condition. Significantly different from vehicle: *p < 0.05, **p < 0.01, ***p < 0.001 in Student’s *t* test. (D,E) COS7 cells expressing GlyT2 were treated with increasing concentrations of ALX1393 (D) or ORG25543 (E) in HBS containing a regular sodium concentration (150 mM NaCl) or low sodium (25 mM NaCl, ChCl substitution) and then subjected to [^3^H]-glycine transport determination.

The docking predicted that, in contrast to ORG25543, ALX1393 binds to the sodium bound in Na1 site. Several pieces of evidence were obtained to confirm this prediction (Figure 7). First, we took advantage of the reversible binding of ALX1393 to the transporter and monitored the decay of the [^3^H]-glycine transport inhibition after 1–4 washes in a medium containing NaCl or choline chloride. As depicted in Figure 7A, the presence of sodium in the washing buffer reduced the washing power, suggesting the ligand was retained at its binding site. As expected, no inhibition decay was observed for ORG25543 according to its irreversibility. A second piece of evidence came from experiments using the A223C mutant of GlyT2 that contains an exogenous cysteine in EL1, a region sensitive to the different conformations of the transporter induced by the substrates during the transport cycle.^44^ As reported previously, the MTSEA-biotin labeling of the A223C mutant is protected by glycine in a buffer containing NaCl but not in a medium containing choline chloride, indicating the accessibility of the cysteine is reduced when sodium is bound (Figure 7B,C). Interestingly, the same behavior was observed in the presence of ALX1393, which abolished the label in NaCl but left it intact in choline chloride. On the contrary, ORG25543 protects the labeling independently of the cation present in the buffer, indicating sodium is not needed for its binding to the transporter. Finally, we performed dose–response curves for the inhibition of [^3^H]-glycine transport by the two compounds in low-sodium conditions (25 mM, choline chloride substitution) as compared to regular sodium concentrations (150 mM NaCl). For the two inhibitors, lowering the external Na^+^ resulted in a reduction of the inhibitory potency measured as an increase in the IC_50_ (Figure 7D,E). However, the IC_50_ showed a higher increase in low sodium for ALX1393 (122.0 ± 20.9 vs 21.8 ± 4.1 nM) than for ORG25543 (51.2 ± 12.3 vs 23.8 ± 7.1 nM), reinforcing the sodium dependence of ALX1393 binding.

The differential interaction of the two inhibitors with GlyT2 TM1 is remarkable. ALX1393 interacts with the cation bound at Na1, and consequently it requires the correct structure of the Na1 site that is importantly contributed by TM1 residues (Tyr-209, Gly-212, Leu-213 and Gly-214). For this reason, the L576I mutant that disrupts the structural Na2 site, in allosteric connection with the Na1 site, prevented the binding of ALX1393 to a much higher extent than that of ORG25543 and was partially resistant to ALX1393 inhibition. This suggests that the basis of the reversible behavior of ALX1393 is its binding to a region that is only structured in the presence of Na^+^.^25^ We found the contacts of ALX1393 with TM1 are mediated by many interactions of slightly lower energy as compared to ORG25543 and seem to be influenced by the presence of the cation. The latter binds directly to a reduced number of TM1 residues, and the binding is not mediated by a Na^+^ ion. The highest energy interaction of ORG25543 with TM1 is Leu-213, a position that could not be tested since its substitution abolishes the activity. However, the reduction of the inhibition by the TM1 mutant W217F exclusively by ORG25543 reflects the higher energy interaction of this compound with TM1. We suggest this is probably the cause of the irreversible binding of ORG25543 to GlyT2. In fact, a chemical derivative of ORG25543 recently generated, compound 1^18^ lacking the −OCH_3_ substituent of ring R5, the main group interacting with TM1 residues, becomes a reversible inhibitor of GlyT2 transport. Interestingly, a photoswitchable derivative of this compound made by substituting the benzyl phenyl ether moiety (that substitutes R6 in ORG25543) by an azobenzene in the *trans* configuration, to which it is structurally homologous, still maintains the reversibility and the noncompetitive inhibition.^44^

On the other hand, our data suggest the interaction of ORG25543 may at least partially involve residues belonging to the S2 allosteric site.^39^ This would explain the selective reduction of ORG25543 potency on the W217F mutant as one of the S2 residues is Trp-217. In addition, our docking predictions and experimental data indicate that ORG25543, in contrast to ALX1393, interacts with TM10 residues (Asp-635 and Ser-640) and probably with Ala-639 backbone, an aspect that could not be confirmed using mutagenesis. Considering that some of these residues are included in the S2 site (Asp-635), it is very likely that the ORG25543 site partially overlaps the allosteric S2 site present in GlyT2. Therefore, although the present experimental approach may not reveal all the interactions of the inhibitors with GlyT2 and perhaps not the complete nature of the binding sites, undoubtedly it allowed us to identify differential structural determinants of GlyT2 for the selective inhibitors ALX1393 and ORG25543. The recently reported structure of GlyT1 in an inward open conformation will certainly assist to get closer to the real GlyT2 structure.^46^

## Conclusions

In this study, we performed the comparative ligand docking of the selective GlyT2 inhibitors ALX1393 and ORG25543 on a validated GlyT2 model based on the dopamine transporter from *Drosophila melanogaster* that includes all GlyT2 ligand sites. Through binding energy calculations on the simulated transporter-ligand complexes and functional analysis of a series of point mutants and cysteine labeling assays, we have revealed some structural determinants of ALX1393 and ORG25543 discrimination by GlyT2. The ligands establish simultaneous contacts with residues present in transmembrane domains 1, 3, 6, and 8 and block the transporter in outward-facing conformation and hence inhibit glycine transport. The dissimilar nature of the interactions with TM1 of ORG25543 and ALX1393 is probably responsible of the irreversible/reversible behavior of the inhibitors. The binding of ORG25543 to TM10 residues suggests a partial overlap of the ligand site with the S2 allosteric site of GlyT2. The differential requirements for the docking and the structural determinants of the inhibitor binding explain some of their individual features and are crucial pieces of information for the design of new reversible inhibitory compounds with suitable pharmacokinetic and pharmacodynamics properties that might be used in pain treatment.

## Methods

### GlyT2 Mutagenesis and Transporter Expression

Substitution mutants were generated with the QuikChange II Site-Directed Mutagenesis kit (Agilent Technologies, Santa Clara, CA, USA), using rGlyT2 or rGlyT1 subcloned in pCDNA3.^47^ The complete coding region of all of the constructs was sequenced to verify that only the desired mutation had been introduced. Plasmids from two independent *Escherichia coli* colonies were expressed in eukaryotic cells as indicated below, and [^3^H]glycine transport and/or immunodetection was performed for verification. COS7 cells were grown and transfected using Turbofect Transfection Reagent (Thermo Fisher Scientific, Waltham, MA, USA), following the manufacturer’s protocol (2 μL reagent/μg of DNA). Cells were incubated for 48 h at 37 °C until used.

### Transport Assays

COS7 cells were washed and incubated at 37 °C in HEPES-buffered saline (HBS, in mM: 150 NaCl, 10 HEPES-Tris, pH 7.4, 1 CaCl_2_, 5 KCl, 1 MgSO_4_, 10 glucose) containing 2 μCi/mL [2-^3^H]glycine (1.6 TBq/mmol; PerkinElmer Life Sciences), at 10 μM final glycine concentration if not otherwise stated.^48^ At the end of the desired time (usually 10 min), reactions were washed and terminated by aspiration. Protein concentration (Bradford) and [2-^3^H]glycine levels (liquid scintillation, LKB 1219 Rackbeta) were determined. Glycine accumulation measured in mock-transfected cells was subtracted from that of the transporter-transfected cells and normalized by the protein concentration. Kinetic analyses were performed by varying glycine concentration in the uptake medium between 0.5 and 500 μM. In inhibition experiments, ALX1393 (Sigma-Aldrich, St. Louis, MO, USA) or ORG25543 (Tocris Bioscience, MN, USA) was added to the transport solution at the indicated concentrations, and the vehicle (DMSO or water, respectively) was added to the control reactions. The determinations of the IC_50_ were performed initially by diluting compounds by log steps with the vehicle from 1 nM to 1 μM. Then, exact IC_50_ values were determined by means of 8-point concentration curves performed over a suitable range using half log dilutions with the vehicle. A nonlinear curve fitting method was used to obtain IC_50_ values using Graphpad Prism software, version 6.0, Graphpad, San Diego, CA, USA.

### Surface Labeling with MTSEA-Biotin and Sulfo-NHS-SS-Biotin

Thiol-specific biotinylation and total surface biotinylation were performed using 2-aminoethylmethanethiosulfonate (MTSEA)-biotin (0.5 mM, Toronto Research Chemicals Inc., Ontario, Canada) and sulfosuccinimidyl-2-(biotinamido)ethyl-1,3-dithiopropionate (sulfo-NHS-SS)-biotin (1 mg/mL, Pierce), respectively, at 4 °C on transfected COS7 cells as described.^13^ After 3 h of incubation with streptavidin-agarose beads (Sigma-Aldrich, St. Louis, MO, USA), reagent-reactive transporter proteins were eluted from the beads with Laemmli buffer [40 mM Tris/HCl (pH 6.8), 2% (w/v) SDS, 10% (v/v) glycerol, 0.1 M DTT (dithiothreitol) and 0.01% bromophenol blue] for 10 min at 70 °C and then analyzed by Western blotting (SDS/7.5% PAGE) with GlyT2-specific antibody.^49^ Protein bands were visualized by enhanced chemiluminescence and quantified on a GS-900 Calibrated Imaging Densitometer using Image Lab software (Bio-Rad Laboratories, Hercules, CA, USA) and film exposures within the linear range. In MTSEA-biotin labeling after cysteine oxidation, COS7 cells expressing the cysteine mutant were treated for 5 min at 22 °C with the preincubation solution containing 100 μM Cu(II)(1,10-phenanthroline)_3_ (CuPh).^50^ The CuPh stock solution (150 mM) was prepared for each experiment by mixing 0.4 mL of 1.25 M 1,10-phenanthroline in water:ethanol (1:1) and 0.6 mL of 250 mM CuSO_4_. Then, the cells were washed and subjected to MTSEA-biotin staining as described above.

### Homology Modeling of GlyT2 and MD Simulations

The experimentally validated equilibrated homology model of GlyT2,^8^ constructed based on the crystallized dopamine transporter from *Drosophila melanogaster* (*d*DAT) (PDB code 4M48)^32^ as the homology model template, was used. Our published GlyT2 homology model was further refined by means of MD simulations^8^ (for the details of methodology, see Supplementary Data).

### Molecular Docking

ALX1393 and ORG25543 found in the Sigma-Aldrich catalogue were obtained as a structure data file (sdf). Two docking softwares were used to ascertain the binding mode of ALX1393 and ORG25543 on the GlyT2 *d*DAT homology model at glycine binding site (S1 site): Autodock Vina^51^ (http://vina.scripps.edu) and Glide.^52^ Ligands preparation and docking methodology are described in Supporting Information.

### Analysis of MD Trajectories

The stability of the complexes (GlyT2-ALX1393 and GlyT2-ORG25543) was evaluated by calculating the RMSD of the Cα atoms along the trajectories, using their starting structures as reference, the RMSF of each residue, and the effective binding free energies between the ligands. The more relevant residues in the binding site were qualitatively estimated using the MM/GBSA^49,50,53,54^ (for the details of methodology, see Supplementary Data).

### Data Analysis

Nonlinear regression fits of experimental transport data and statistical analysis were performed with GraphPad Prism 6.01 (San Diego, CA, USA). Bars represent SEM of at least triplicate determinations. The representative experiments shown were repeated at least three times with equivalent results.

## Supporting Information Available

Structural and energetic stability of the poses; main features of the analyzed mutants; supplementary computational methods; and supplementary references (PDF)

## Abbreviations

DAT: dopamine transporter
GlyT: glycine transporter
MD: molecular dynamics
MTSEA: 2-aminoethylmethanethiosulfonate
NSS: neurotransmitter:sodium symporters
RMSD: root-mean-square deviation
RMSF: root-mean-square fluctuation
TM: transmembrane domain
