# Supplementary material for: Structural Determinants of the Neuronal Glycine Transporter 2 for the Selective Inhibitors ALX1393 and ORG25543
Source: ACS Chem Neurosci. 2021 May 18;12(11):1860–72. doi: 10.1021/acschemneuro.0c00602 (PMC8691691; doi:10.1021/acschemneuro.0c00602)
Supplement: Supplementary file 1 — cn0c00602_si_001.pdf [file cn0c00602_si_001.pdf]

Structural determinants of the neuronal glycine transporter 2 (GlyT2) for the selective inhibitors ALX1393 and ORG25543

Cristina Benito-Muñoz[1,2], Almudena Perona[3], Raquel Felipe[1,2], Gonzalo Pérez-Siles[1,2], Enrique Núñez [1,2], Carmen Aragón[1,2,4] and Beatriz López-Corcuera[1,2,4]*

[1]*Departamento de Biología Molecular Universidad Autónoma de Madrid. Spain.*
[2]*Centro de Biología Molecular "Severo Ochoa" Consejo Superior de Investigaciones Científicas-Universidad Autónoma de Madrid. Spain.*
[3]*Departamento de Química en Ciencias Farmacéuticas, Universidad Complutense de Madrid, Madrid, Spain.*
[4]*IdiPAZ-Hospital Universitario La Paz, Universidad Autónoma de Madrid, Spain*

Supporting information

1. Structural and energetic stability of the poses

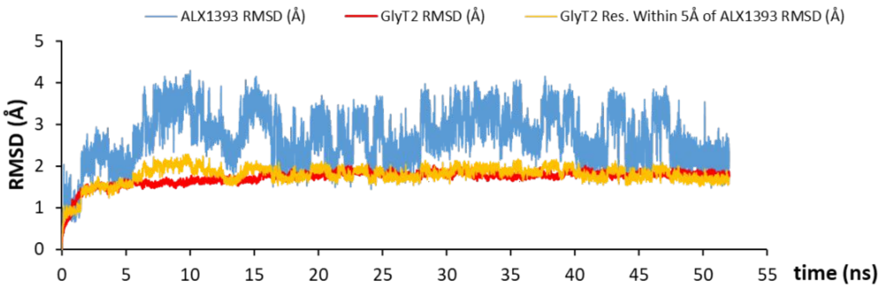

Figure S1A. RMSD values relatives to the starting structure along 50 ns of MD simulation of GlyT2 complexed to ALX1393. The Y axis shows the RMSD values in Å and the X axis the time in nanoseconds. RMSD values are represented in red for the protein GlyT2, orange for the residues of GlyT2 within 5 Å of ALX 13933 and in blue for ALX 1393.

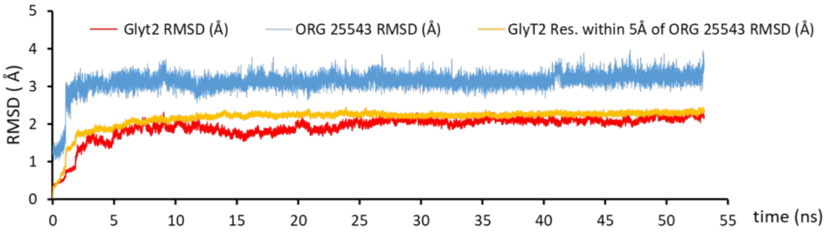

Figure S1B. RMSD values relatives to the starting structure along 50 ns of MD simulation of GlyT2 complexed to ORG25543. The Y axis shows the RMSD values in Å and the X axis the time in nanoseconds. RMSD values are represented in red for the protein GlyT2, orange for the residues of GlyT2 within 5 Å of ORG 25543 and in blue for ORG 25543.

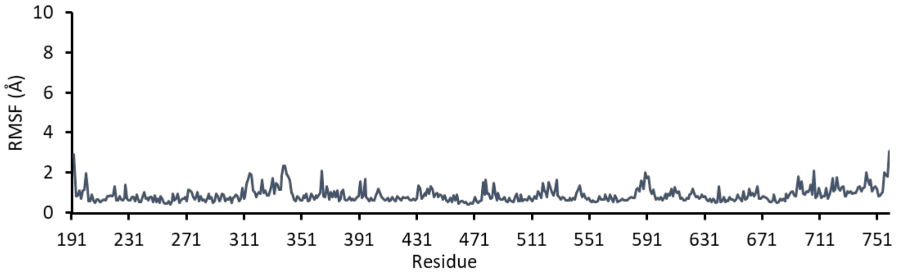

Figure S2A. Average fluctuations of the residues of GlyT2-ALX1393 complex during the simulation. Y axis represents Cα RMSD in Å and X axis, residue number of GlyT2.

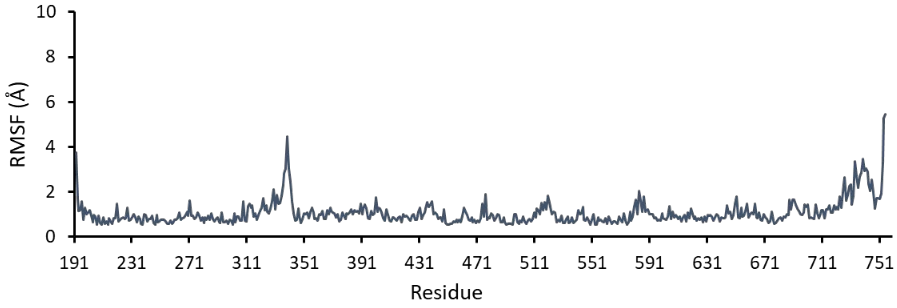

Figure S2B. Average fluctuations of the residues of GlyT2-ORG25543 complex during the simulation. Y axis represents Cα RMSD in Å and X axis, residue number of GlyT2.

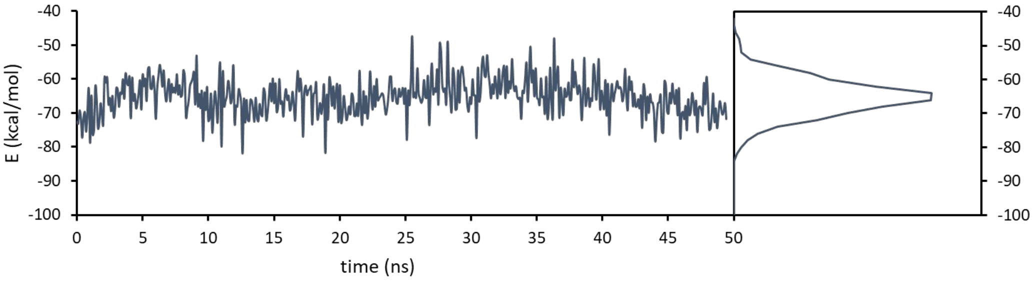

Figure S3A. 2D free energy plot for GlyT2-ALX 1393 complex. The black line shows the energy value at each step of the simulation. The X axis shows time in nanoseconds, the left Y axis shows global energy values (kcal/mol), and the right Y axis shows the density energy values achieved by the complex during the simulation.

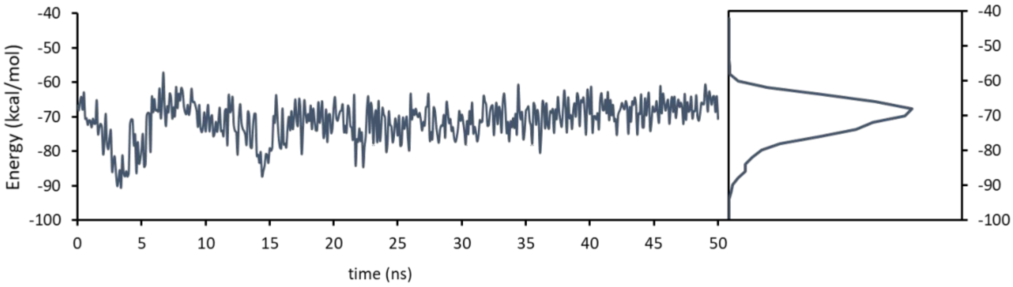

Figure S3B. 2D free energy plot for GlyT2-ORG 25543 complex. The black line shows the energy value at each step of the simulation. The X axis shows time in nanoseconds, the left Y axis shows global energy values (kcal/mol), and the right Y axis shows the density energy values achieved by the complex during the simulation.

(A)

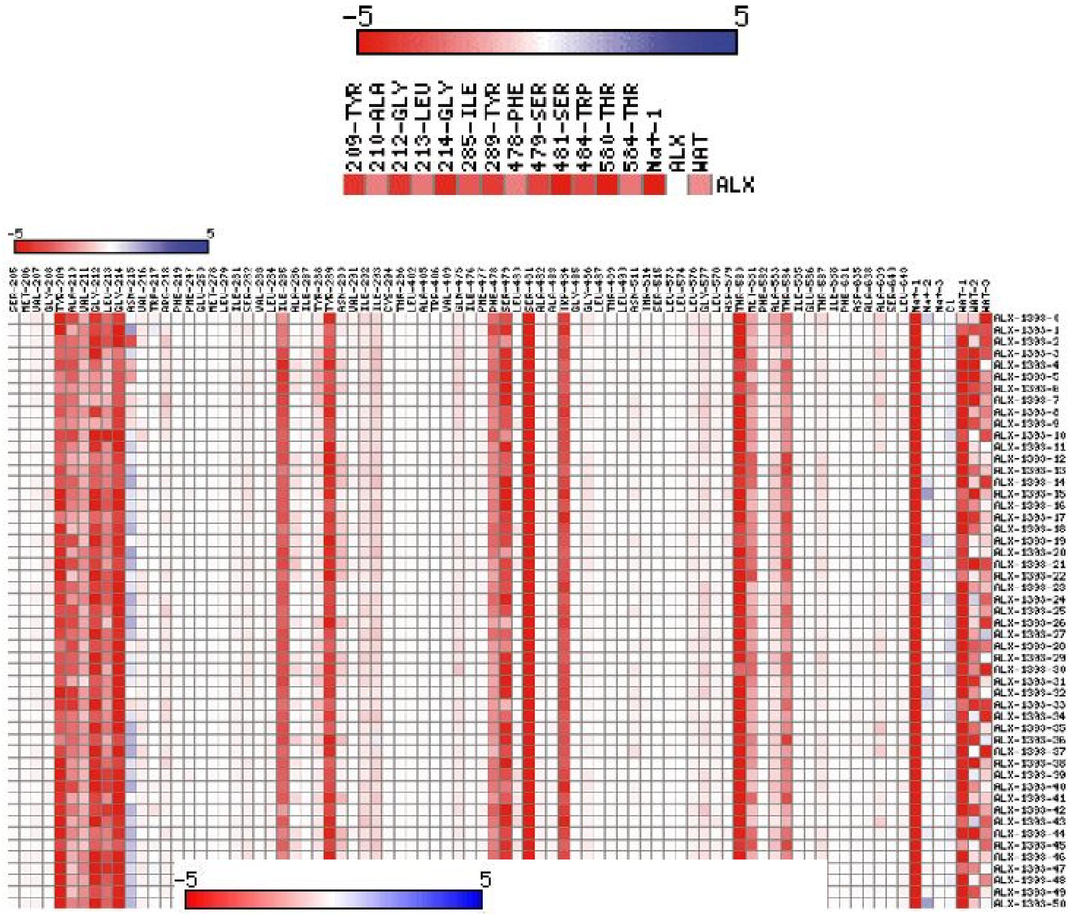
-5
5
209-TYR
210-ALA
212-GLY
213-LEU
214-GLY
285-ILE
289-TYR
478-PHE
479-SER
481-SER
484-TRP
580-THR
584-THR
Na+-1
ALX
WAT
ALX

(B)

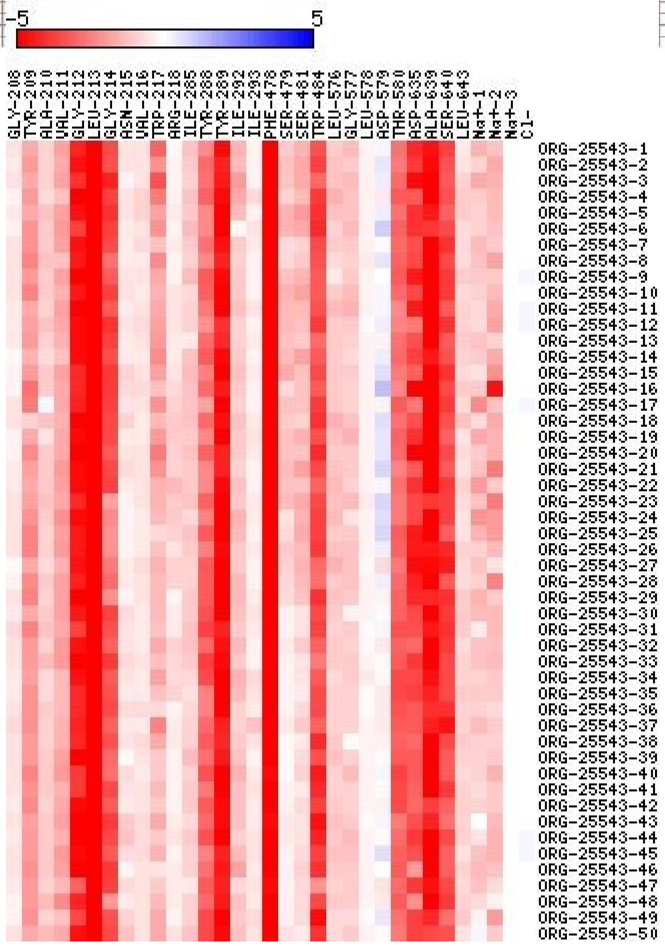
-5
5
GLY-208
TYR-209
ALA-210
VAL-211
GLY-212
LEU-213
GLY-214
ASN-215
VAL-216
TRP-217
ARG-218
ILE-285
TYR-288
THR-289
ILE-292
ILE-293
PHE-478
SER-479
SER-481
TRP-484
LEU-576
GLY-577
LEU-578
ASP-579
THR-580
ASP-635
ALA-639
SER-640
LEU-643
Na+-1
Na+-2
Na+-3
Cl-
ORG-25543-1
ORG-25543-2
ORG-25543-3
ORG-25543-4
ORG-25543-5
ORG-25543-6
ORG-25543-7
ORG-25543-8
ORG-25543-9
ORG-25543-10
ORG-25543-11
ORG-25543-12
ORG-25543-13
ORG-25543-14
ORG-25543-15
ORG-25543-16
ORG-25543-17
ORG-25543-18
ORG-25543-19
ORG-25543-20
ORG-25543-21
ORG-25543-22
ORG-25543-23
ORG-25543-24
ORG-25543-25
ORG-25543-26
ORG-25543-27
ORG-25543-28
ORG-25543-29
ORG-25543-30
ORG-25543-31
ORG-25543-32
ORG-25543-33
ORG-25543-34
ORG-25543-35
ORG-25543-36
ORG-25543-37
ORG-25543-38
ORG-25543-39
ORG-25543-40
ORG-25543-41
ORG-25543-42
ORG-25543-43
ORG-25543-44
ORG-25543-45
ORG-25543-46
ORG-25543-47
ORG-25543-48
ORG-25543-49
ORG-25543-50

Figure S4. Interaction matrix of GlyT2-ligand complexes over the 50 ns. (A) GlyT2-ALX1393 interaction matrix. (B) GlyT2-ORG25543 interaction matrix.

2. *Main features of the analyzed mutants*

Table S1: activity of the generated mutants.

Mutant	Activity (% of wtGlyT2)	Expression (% of wtGlyT2)	EC_{50} Gly (µM)	IC_{50} [(a)] ALX1393	IC_{50} [(a)] ORG25543
wtGlyT2	100 ± 9	100 ± 31	120 ± 26	31 ± 6	18 ± 5
Y209F/S/T	KO				
A210C	67 ± 6	107± 23	336 ± 10	23 ± 3	20 ± 12
L213C	KO				
G214L	84 ± 33	67± 18	78 ± 12	38 ± 3	17 ± 10
W217F	63 ± 8	85 ± 29	245 ± 27	23 ± 5	93 ± 7
I285F	58 ± 13	146 ± 37	603 ± 86	91 ± 6	15 ± 4
Y289C/S/T	KO				
I292C	64 ± 15	73 ± 15	176 ± 15	36 ± 4	24 ± 3
F478Y	60 ± 12	100 ± 25	290 ± 13	50 ± 5	19 ± 7
S479A	KO				
S481G	42 ±11	70 ± 10	149 ± 11	75 ± 8	>10 [(b)]
W484C	9 ± 4	72 ± 18	2338 ± 209	>10	(c)
W484F	11 ± 2	69 ± 13	910 ± 83	>10	>10
L576I	76 ± 20	96 ± 15	372 ± 40	101 ± 22	111 ± 15
T580C	26 ± 6	41± 12	600 ± 81	84 ± 9	96 ± 10
T584F	73 ± 21	53 ± 9	180 ± 0.6	>10	>10
T584L	35 ± 21	42 ± 8	1200 ± 189	>10	>10
D635E	15 ± 3	105 ± 16	123 ± 6	30 ± 4	>10
A639C	55 ± 20	78 ± 26	176 ± 14	22 ± 4	51 ± 2
S640C	27 ± 9	85 ± 5	111 ± 2	30 ± 7	130 ± 6

(a) Conditions of glycine concentrations as in Figs. 4 and 5.

(b) >10, IC_{50} values at least 10 times higher than that of wtGlyT2.

(c) ND: not determined, insufficient transport activity to precisely calculate an IC_{50}.

3. *Supplementary Computational Methods*

Homology modelling of GlyT2- The experimentally validated equilibrated homology model of GlyT2 (1), constructed based on the crystallized dopamine transporter from *Drosophila melanogaster* (*d*DAT) (PDB code 4M48) (2) as the homology model template, was used. Alignments between the sequence of *d*DAT and GlyT2 were obtained by profile-profile alignment using the program Muscle (3). Profiles for the protein sequence were obtained by multiple sequence alignment (MSA), using the program MAFFT (4). Sequences included in MSAs were detected by the program blastp (5) against the NCBI's nr database, with an e-value below 1e-15. A *h*GlyT2 model with the bound substrate (glycine) and coupled ions was obtained using MODELLER version 9.13 (6). Initial alignments were iteratively refined based on the evaluation of the obtained model. The modelled region for GlyT2 was from Lys-191 to Trp-760, excluding the loop between Leu-329 and Ala-365, which is not modelled for *d*DAT.

Molecular dynamics simulations- Our published GlyT2 homology model was further refined by means of molecular dynamics simulations as described (1). Using the Membrane Builder module (7) (8) in CHARMM-GUI (www.charmm-gui.org) (9), each protein was inserted in a pre-equilibrated box containing a POPC lipid bilayer, POPE lipid bilayer, cholesterol molecules (in a proportion 2:2:1) surrounded by water, and a 0.15 M concentration of Na^+ and Cl^- ions. We performed molecular dynamics simulations using AMBER (10), the protocol was as follows: (i) energy minimization to relax the initial model (20 ps), (ii) heating (100k), holding lipid molecules and Cα carbons fixed (100 ps), (iii) heating (300k), holding lipid molecules and Cα carbons fixed (100 ps), (iv) holding, to equilibrate the water molecules around the solutes (10 x 200 ps, lowering restraints), (v) production at constant temperature and pressure (30 ns). All the simulations were performed at constant pressure (1 atm) and temperature (300 °K) with an integration time step of 2 fs. The SHAKE algorithm (11) was used to constrain all the bonds involving H atoms at their equilibrium distances. Periodic boundary conditions and the particle mesh Ewald methods were applied to treat long-range electrostatic effects (12). AMBER ff03 (13), Lipid11 (14), Lipid14 (15) and TIP3P (16) force fields were used in all cases. All the trajectories and analysis were performed using the AMBER 12 computer program and associated modules (10). Mutants were built using the Mutagenesis Wizard implemented in PyMol (The PyMOL Molecular Graphics System).

Molecular docking- ALX1393 and ORG25543 found in the Sigma-Aldrich catalogue were obtained as a structure data file (sdf). The sdf were transformed into 3D structures via LigPrep; tautomer creation. Partial charge calculation, and energy minimization were conducted using the OPLS_2005 force field. The OPLS_2005 parameters are described in (17). The GlyT2 homology model has included three Na^+ ions and one Cl^-ion. Two docking softwares were used to ascertain the binding mode of ALX1393 on the GlyT2 *d*DAT homology model at glycine binding site (S1 site). Autodock Vina, adopting a basic docking protocol, flexible ligand and rigid receptor (18) (http://vina.scripps.edu), and ligand and protein flexible docking with Glide using XP GlideScore as scoring function to rank-order compounds (19). The most favourable poses were selected according to its predicted docking energy. The resultant complexes were used as the starting point for molecular dynamics studies, following the same protocol describe above. The representative structure of the most populated cluster along the simulation (50ns MD simulations) were chosen as the final poses of GlyT2 bound to the inhibitors using cluster analysis with CPPTRAJ (AMBER 12, (10).

Analysis of MD trajectories. The stability of the complexes (ALX1393-GlyT2 and ORG25543-GlyT2) were evaluated by calculating the root-mean-square-deviation (RMSD) of the Cα atoms along the trajectories, using their starting structures as reference. Additionally, the root-mean-square-fluctuation (RMSF) of each residue, relative to the corresponding average value, was calculated once each snapshot had been fitted to its initial structure. The effective

binding free energies between the ligands and the more relevant residues in the binding site were qualitatively estimated using the MM/GBSA (20,21). MM/GBSA is a popular approach to estimate the free energy of the binding of small ligands to biological macromolecules. MM/GBSA takes into account a MM interaction term, a solvation contribution thorough a generalized born (GB) model, and a surface area (SA) contribution to account for the non-polar part of desolvation. The MM part estimates the enthalpic contributions for the protein–ligand interactions (bonded, electrostatic, vanderWaals). The polar solvation energy represents the electrostatic interaction between the solute and the continuum solvent. In addition, three non-polar solvation terms include cavitation, dispersion, and repulsion energies, representing the cost of making a cavity in the solvent, as well as the attractive and repulsive parts of the van der Waals interactions between the solute and the solvent. All these three non-polar solvation terms are free energies and in particular the cavitation energy should have important entropic components, representing the reorganization of the solvent around the solute. In summary, in MM/GBSA, the free energy of a state is estimated from the following sum: G = Ebnd + Eel + EwdW + Gpol + Gnp - TS. A 12–6 Lennard-Jones term was used to model de MM contribution. For GB, the solute dielectric constant was set to four while that of the solvent was set to 80, and the dielectric boundary was calculated using a solvent probe radius of 1.4 Å. The polar contribution is calculated using GB, and the non-polar energy is estimated by solvent accessible surface area (SASA).

Supplementary references

1. Benito-Muñoz C, Perona A, Abia D, dos Santos HG, Núñez E, Aragón C, et al. Modification of a Putative Third Sodium Site in the Glycine Transporter GlyT2 Influences the Chloride Dependence of Substrate Transport. Front Mol Neurosci. 2018 Sep;11(347).

2. Penmatsa A, Wang KH, Gouaux E. X-ray structure of dopamine transporter elucidates antidepressant mechanism. Nature. 2013 Nov 7;503(7474):85–90.

3. Edgar RC. MUSCLE: multiple sequence alignment with high accuracy and high throughput. Nucleic Acids Res. 2004;32:1792–7.

4. Katoh K. MAFFT: a novel method for rapid multiple sequence alignment based on fast Fourier transform. Nucleic Acids Res. 2002;30:3059–66.

5. Altschul SF. Basic local alignment search tool. J Mol Biol. 1990;215:403–10.

6. Sali A. Comparative protein modelling by satisfaction of spatial restraints. J Mol Biol. 1993;234:779–815.

7. Jo S. Automated builder and database of protein/membrane complexes for molecular dynamics simulations. PLoS One. 2007;2(e880).

8. Jo S. CHARMM-GUI Membrane Builder for mixed bilayers and its application to yeast membranes. Biophys J. 2009;97:50–8.

9. Jo S. CHARMM-GUI: a web-based graphical user interface for CHARMM. J Comput Chem. 2008;29:1859–65.

10. D. A. Case TAD. AMBER 12. Univ Calif F. 2012;

11. Ryckaert J-P. Numerical integration of the cartesian equations of motion of a system with constraints: Molecular dynamics of n-alkanes. J Comput Phys. 1977;23:327−341.

12. Darden T. Particle mesh Ewald: An N·log(N) method for Ewald sums in large systems. J Chem Phys. 1993;98:10089–92.

13. Duan Y. A point-charge force field for molecular mechanics simulations of proteins based on condensed-phase quantum mechanical calculations. J Comput Chem. 2003;24:1999–2012.

14. Skjevik AA. LIPID11: a modular framework for lipid simulations using amber. J Phys Chem B. 2012;(116):11124–36.

15. Dickson CJ. Lipid14: The Amber Lipid Force Field. J Chem Theory Comput. 2014;10:865–79.

16. Jorgensen WL. Comparison of simple potential functions for simulating liquid water. J Chem Phys. 1983;(79):926–35.

17. Banks JL; B. Integrated Modeling Program, Applied Chemical Theory (IMPACT). J Comp Chem. 2005;26(1752).

18. Trott O. AutoDock Vina: improving the speed and accuracy of docking with a new scoring function, efficient optimization, and multithreading. J Comput Chem. 2010;31:455–61.

19. Schrödinger LLC. Small-Molecule Drug Discovery Suite 2016-3: Glide, version 7.2,. N Y NY. 2016;

20. Miller BR, McGee TD, Swails JM, Homeyer N, Gohlke H, Roitberg AE. MMPBSA.py: An Efficient Program for End-State Free Energy Calculations. J Chem Theory Comput. 2012 Sep 11;8(9):3314–21.

21. Genheden S, Ryde U. The MM/PBSA and MM/GBSA methods to estimate ligand-binding affinities. Expert Opin Drug Discov. 2015;10(5):449–61.
